# Alleghany County Medical Society

**Published:** 1879-10

**Authors:** 


### ALLEGHANY COUTY MEDICAL SOCIETY.

---

The regular monthly meeting of the Alleghany County Medical Society was held in Cumberland, Md., August 19th, 1879. Dr. James M. Porter, President, occupied the chair and the following members were present : Drs. G. B. Fundenberg, M. A. Carr, W. H. McCormick, E. H. Parsons, J. W. Englar, J. A. Doerner, S. P. Smith, C. H. Ohr, T. M. Healey, and O. M Schindel, Secretary.

Dr. Ohr, Chairman of Committee on the Revision of the Constitution, said they were ready to report a constitution which he read to the Society in detail, and upon motion it was unanimously adopted.

Dr. Ohr presented to the Society, a patient of Dr. W. J. Pipers, with chronic disease of the knee joint, in a boy thirteen years old; the disease had existed for about fifteen months, was the result of a sprain. At this time the joint is very much swollen, and there is a fistulous track opening into the joint, the leg below the knee is very much atrophied, partial anchylosis exists. There is a history of cancer in the boy's family, father having died with it. Dr. Fundenberg thought the case an unfavorable one, but advised liberating the pus, and washing out the joint freely with a dilute solution of carbolic acid, making hyper distention by this means and with the use of compressed sponge over the joint healthy granulations; but if these means failed, amputation as a dernier resort. Dr. Ohr was of the opinion there was extensive destruction of the cartilages of the joint, and the cancellated epiphyses of the bones were involved in the softening process which was going on, and not much could be expected from an attempt at resection of the joint, but would wait a reasonable time and use rational means, and if no improvement took place would amputate. Dr. Schindel would apply "Esmarch's bandage" and attempt resection, but if upon opening the joint the bones were found involved to any serious extent, would proceed and finish the operation by an amputation.

Dr. J. M. Porter reported a case of puerperal convulsions which had occurred in his practice. The patient was a well developed woman, a primipara. At about the end of the eighth month of pregnacy the patient had three convulsions before the doctor saw her. Found the legs œdematous and mottled in appearance, the os-uteri entirely closed and no labor pains. Puting the patient under chloroform pains soon set in, used manual dilatation, and in a couple of hours a living child was delivered by the use of the instrument. Patient had nine convulsions before and seven after the delivery; after the delivery, discontinued the use of the chloroform, and used hypodermic injections of morphia. Patient recovered, but there was temporary paralysis of the muscles of the left side of the face which lasted for one week.

Dr. Fundenberg advocated the bringing on of labor in such cases by manual efforts at dilatation; would have bled this patient as well as used chloroform; recommended in the highest terms the hypodermic injections of morphia after the delivery; had found them to have the happiest effect in controlling the convulsions.

Dr. Ohr said he should have used the lancet unsparingly in this case, contending that the lancet was one of the best known means of causing rapid dilatation of the os-uteri, and of protecting the brain from undue blood pressure, and as this was the organ most likely to be injured thought our first consideration should be to protect it. Said the hypodermic injection of a half grain of pilocarpin would produce prompt dilatation of the os; after delivery there was no agent equal to the hypodermic injections of morphia in controlling the convulsions.

Dr. Fundenberg offered a resolution, which was passed by the Society unanimously, requesting the editors of local papers not to mention the name of physicians in connection with any medical or surgical cases, as is their customs, and the practice of some of the members of this Society, and it was ordered that the secretary of the Society present these resolutions to each member of the Society for his signature, and those who refuse to sign them to be reported at the next meeting, so that they may know who the persons are that indulge in these breaches of medical ethics, and that they may reach them through the society.

The mortuary report for Cumberland for the past month shows that thirteen deaths have occurred—adults eight, children five—resulting from the following causes: Intestinal catarrh, one, chronic diarrhœa, one, gastro-enteritis, one, heart disease, one, cholera infantum, two, general exhaustion, two, inanition, one, typhoid pneumonia, one, typhoid fever, one, abdominal tumor, one, general dropsy, one.

After a very interesting and harmonious meeting, the society adjourned to meet at the regular time and place next month.—*Maryland Medical Journal.*

